# The dilemma of ^18^F-FDG PET/CT thyroid incidentaloma: what we should expect from FNA. A systematic review and meta-analysis

**DOI:** 10.1007/s12020-021-02683-4

**Published:** 2021-03-24

**Authors:** Lorenzo Scappaticcio, Arnoldo Piccardo, Giorgio Treglia, David N. Poller, Pierpaolo Trimboli

**Affiliations:** 1 Unit of Endocrinology and Metabolic Diseases, University of Campania ‘L. Vanvitelli’’, Naples, Italy; 2 Department of Nuclear Medicine, Galliera Hospital, Genoa, Italy; 3 Department of Nuclear Medicine and Molecular Imaging, Lausanne University Hospital, Lausanne, Switzerland; 4 Faculty of Biology and Medicine, University of Lausanne, Lausanne, Switzerland; 5 Clinic of Nuclear Medicine and Molecular Imaging, Imaging Institute of Southern Switzerland, Ente Ospedaliero Cantonale, Bellinzona, Switzerland; 6 Health Technology Assessment Unit, Academic Education, Research and Innovation Area, Ente Ospedaliero Cantonale, Bellinzona, Switzerland; 7 Departments of Cytology & Pathology, Queen Alexandra Hospital, Portsmouth, UK; 8 UCL Cancer Institute, 72 Huntley St., Bloomsbury, London, WC1E 6DD UK; 9 Clinic for Endocrinology and Diabetology, Lugano Regional Hospital, Ente Ospedaliero Cantonale, Lugano, Switzerland; 10 Faculty of Biomedical Sciences, Università della Svizzera Italiana (USI), Lugano, Switzerland

**Keywords:** Thyroid cancer, ^18^F-FDG, PET/CT incidentaloma, FNA, Bethesda

## Abstract

**Purpose:**

^18^F-FDG thyroid incidentaloma (TI) occurs in ~2% of PET/CT examinations with a cancer prevalence of up to 35–40%. Guidelines recommend fine-needle aspiration cytology (FNA) if a focal ^18^F-FDG TI corresponds to a sonographic nodule >1 cm. The aim of this systematic review and meta-analysis was to provide evidence-based data on the diagnostic distribution of ^18^F-FDG TIs in the six Bethesda systems for reporting thyroid cytopathology (BETHESDA) subcategories.

**Methods:**

Original studies reporting ^18^F-FDG TIs and cytologically classified according to BETHESDA were included. Six separate meta-analyses were performed to obtain the pooled prevalence (95% confidence interval, 95% CI) of ^18^F-FDG TIs in the six BETHESDA subcategories.

**Results:**

Fifteen studies were finally included. Nine studies were from Asian/Eastern and six from Western countries. FNA data according to BETHESDA was available in 2304 cases. The pooled prevalence of ^18^F-FDG TIs according to BETHESDA was BETHESDA I 10% (6–14), BETHESDA II 45% (37–53), BETHESDA III 8% (3–13), BETHESDA IV 8% (5–12), BETHESDA V 6% (4–9), BETHESDA VI 19% (13–25). A significantly different prevalence was found in the BETHESDA IV between Asian/Eastern (2%) and Western (19%) studies.

**Conclusion:**

Two-thirds of focal ^18^F-FDG TIs undergoing FNA have either malignant (BETHESDA VI) or benign (BETHESDA II) cytology while a minority will have indeterminate (BETHESDA III or IV) FNA results. Significant differences between Asian/Eastern and Western studies are also present in the prevalence of indeterminate FNA results.

## Introduction

The advent in recent years of high-performance medical imaging tools, such as ultrasonography (US), computed tomography (CT), magnetic resonance (MRI), and positron emission tomography/computed tomography (PET/CT) with various tracers such as fluorine-18-fluorodeoxy-glucose (^18^F-FDG), radiolabelled choline, and radiolabelled prostate-specific membrane antigen, has improved the management of patients [1, 2]. However, using these new imaging modalities has led to the frequent detection of unexpected asymptomatic lesions, “incidentalomas”: an increasingly important topic in clinical practice [3].

Because of the high incidence of nodular thyroid disease in the general population, the identification of thyroid incidentalomas (TI) occurs frequently in clinical practice [4, 5]. According to evidence-based data, the prevalence of ^18^F-FDG TI is about 2–3% of all PET/CTs, approximately two in three showing focal uptake [5]. Focal ^18^F-FDG TI is defined as any focal uptake corresponding to a given thyroid nodule. The cancer detection rate in focal ^18^F-FDG TIs is reported to be up to 35–40% [5–7].

The 2015 American Thyroid Association guidelines for adult patients with thyroid nodules and differentiated thyroid cancer recommend performing fine-needle aspiration cytology (FNA) in all ^18^F-FDG PET/CT TIs with a sonographically confirmed thyroid nodule >1 cm [8]. Although US is the key diagnostic modality in the initial diagnostic assessment of thyroid nodules, the diagnostic accuracy and performance of thyroid imaging reporting and data systems (TIRADSs) in ^18^F-FDG TI needs to be further validated [9]. Moreover, significant selection bias may influence the calculation of the risk of malignancy (ROM) of ^18^F-FDG TIs for several reasons. Firstly, the majority of studies of ^18^F-FDG TI only report outcomes for nodules undergoing thyroid diagnostic work-up, which represent a minority of most institutional series of patients. Oncological patients with ^18^F-FDG TI have a low likelihood of thyroid surgery if the comorbid non-thyroidal malignancy is more aggressive and/or of worse prognosis [10]. Secondly, most studies use histopathological assessment as the reference standard for malignant lesions and non-neoplastic FNA cytology as the reference standard for benign lesions as benign lesions are often not operated [11]. Thirdly, ^18^F-FDG TIs with indeterminate FNA, which is the Bethesda system for reporting thyroid cytopathology [12] subcategories III and IV, without histological diagnoses, are generally not included in statistical analyses of ROM even if the expected ROM is not negligible.

In view of the above, before undertaking FNA in an ^18^F-FDG TI, it is important to know the relative frequency of the BETHESDA cytological subcategories in an ^18^F-FDG TI. For all thyroid nodules a recently published meta-analysis by Vuong et al. [13]. shows pooled BETHESDA frequencies as follows: Category I non-diagnostic 12.2%, Category II benign 62.3%, Category III AUS/FLUS 8.0%, Category IV follicular neoplasm/suspicious for follicular neoplasm 6.1%, Category V suspicious for malignancy 3.7%, and Category VI malignant 7.4%. The purpose of the current study was to ask the question—are the cytologic findings in ^18^F-FDG TI different from those in non-FDG PET/CT detected thyroid nodules? It is known for example that Hürthle cell/oncocytic thyroid lesions (HTL) may be over-represented in ^18^F-FDG avid thyroid nodules [14]. With this information, the clinician can better manage ^18^F-FDG TI patients using FNA, particularly for HTL. Both benign and malignant HTLs are known to be ^18^F-FDG avid [15]. However, HTL usually falls into class IV of BETHESDA [12], where the resection rate was reported 60.5% and ROM 28.9% [13].

This study was designed to achieve evidence-based information on the relative distribution of ^18^F-FDG TIs in the various cytological categories of BETHESDA (non-diagnostic, benign, atypia of undetermined significance or follicular lesion of undetermined significance, follicular neoplasm/suspicious of a follicular neoplasm, suspicious for malignancy, and malignant) [12], providing information useful for the clinical management of ^18^F-FDG TI.

## Methods

### Guidelines followed

In this study, all procedures utilized were consistent with PRISMA guidelines [16].

### Search strategy

Three investigators (LS, AP, and PT) independently conducted a comprehensive literature search of online databases MEDLINE (PubMed) and Scopus using the following search terms and their combinations: thyroid, nodule, incidentaloma, FDG, PET, positron. A commencement date limit was not used. The last search was carried out on 31 October 2020. No language restrictions were imposed. The search was restricted to human studies. Three investigators (LS, AP, and PT) screened independently titles and abstracts of the retrieved articles, reviewed the full-texts, and then selected articles for inclusion. References from included studies were also screened for additional articles.

### Eligibility criteria

The major inclusion criterion was original studies reporting ^18^F-FDG TIs undergoing FNA and classified according to BETHESDA. The following studies were excluded: (1) with an overlapping patient or nodule data; (2) reporting only some BETHESDA cytologic subcategories (because these results did not allow calculation of the frequency of each cytological subclass of BETHESDA); (3) with ≤10 ^18^F-FDG TIs. Three researchers (LS, AP, and PT) applied the above criteria selecting studies for inclusion. Disagreements were resolved via online consensus discussion among all the authors.

### Data extraction

For the included studies, the following data were extracted independently and coded in duplicate by three investigators (LS, AP, and PT), in the pilot form: (a) author, publication year, country, study design; (b) number of PET scans performed during the study period; (c) patients’ ages and gender; (d) SUVmax value; (e) size of ^18^F-FDG TIs; (f) number of ^18^F-FDG TI undergoing FNA; (g) number of ^18^F-FDG TI in all six BETHESDA categories; (h) number of ^18^F-FDG TI with a histological diagnosis. The collated details were cross-checked and any discrepancies were fully reconciled by joint re-evaluation among the authors.

### Study quality assessment

The risk of bias of the included studies was assessed independently by three investigators (LS, AP, and PT) using the National Heart, Lung, and Blood Institute Quality Assessment Tool (https://www.nhlbi.nih.gov/health-topics/study-quality-assessment-tools).

### Data analysis

The characteristics of included studies were summarized. A proportion meta‐analysis calculation was used to obtain the pooled rate of ^18^F-FDG TI assessed in all BETHESDA categories [12]. Six separate meta-analyses were performed to obtain the pooled prevalence (95% confidence interval, 95% CI) of ^18^F-FDG TIs in the six different BETHESDA categories. Heterogeneity between studies was assessed by using *I*^2^, with 50% or higher values regarded as high heterogeneity. If the presence of heterogeneity was identified, further analyses were performed to explain it. The Egger’s test was carried out to evaluate the possible presence of significant publication bias. For statistical pooling of data, a random-effect model was used. A *p* < 0.05 was regarded as significant. All analyses were performed using StatsDirect statistical software (StatsDirect Ltd; Birkenhead, Merseyside, UK).

## Results

### Study selection

**Fig. 1:**
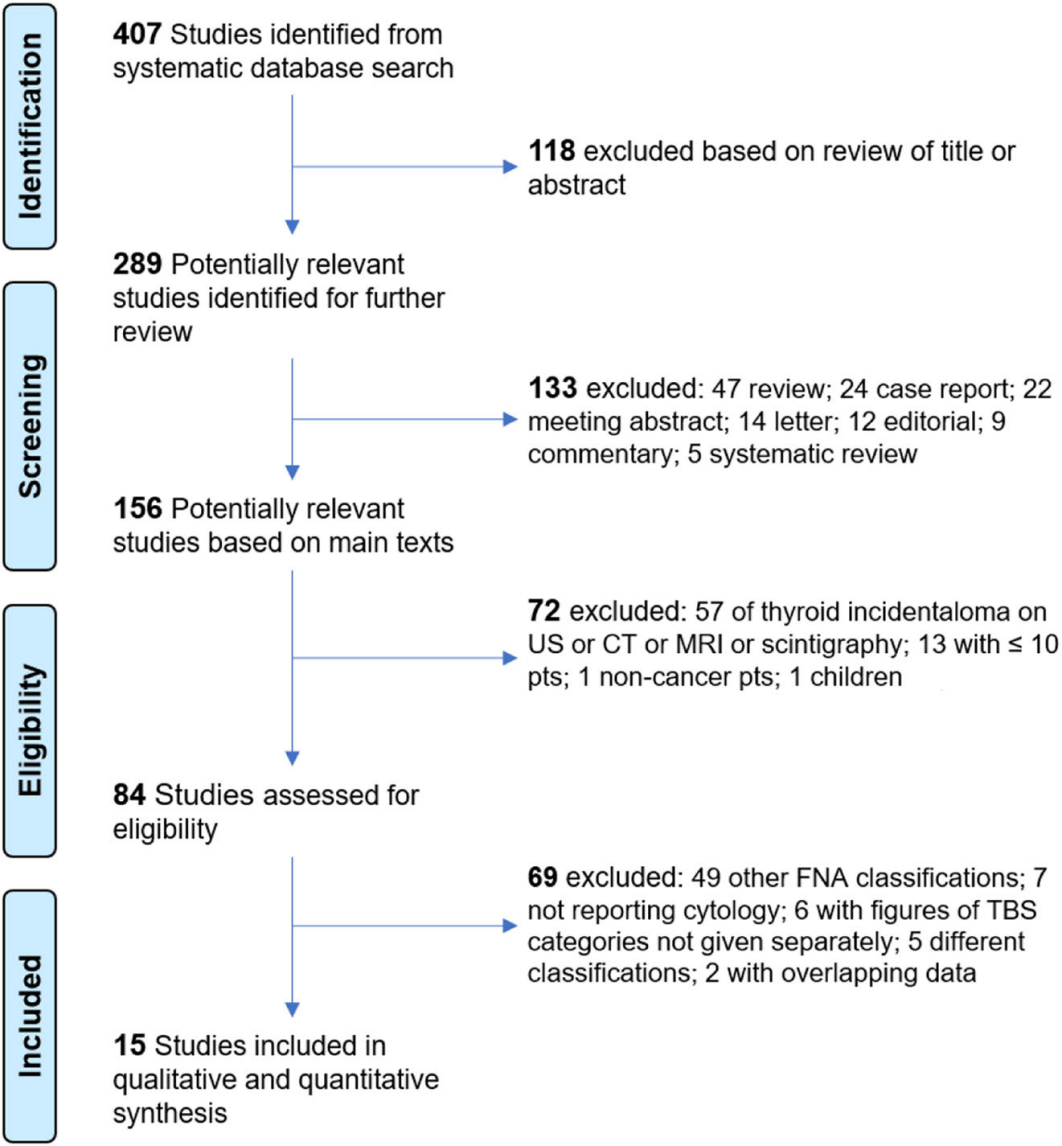
Diagram of the flow of searching data. US ultrasound, CT computed tomography, MRI magnetic resonance imaging, pts patients, FNA fine-needle aspiration. Bethesda system for reporting thyroid cytopathology (BETHESDA)

Literature searches using the above algorithms yielded 407 studies. All the records were assessed as depicted in Fig. 1. Of these, 289 were screened, 84 were assessed as eligible, and 15 [17–31] were included in the final systematic review and meta-analysis.

### Study quality assessment

Table 1 summarizes the quality assessment of the 15 included studies. The risk of bias for each study was judged as low for 12 items. All studies were high risk with respect to sample size. None of the studies reported power or sample size justification.

**Table 1:** Risk of bias summary: review authors' judgements on the quality of the 15 included studies

First author	Lee [17]	Kim [18]	Choi [19]	Lee [20]	Jamsek [21]	Kim [22]	Kim [23]	Yoon [24]	Hagenimana [25]	Li [26]	Suh [27]	Thuillier [28]	De Guevara [29]	Kaliszewski [30]	Kamakshi [31]
1. Research question.	L	L	L	L	L	L	L	L	L	L	L	L	L	L	L
2. Study population.	L	L	L	L	L	L	L	L	L	H	L	L	L	L	L
3. Participation rate.	NR	NR	NR	NR	NR	NR	NR	NR	NR	NR	NR	NR	NR	NR	NR
4. Groups recruiting.	L	L	L	L	L	L	L	L	L	L	L	L	L	L	L
5. Sample size.	H	H	H	H	H	H	H	H	H	H	H	H	H	H	H
6. Exposure assessment	L	L	L	L	L	L	L	L	L	L	L	L	L	L	L
7. Sufficient timeframe to see an effect.	L	L	L	L	L	L	L	L	L	L	L	L	L	L	L
8. Different levels of exposure of interest.	L	L	L	L	L	L	L	L	L	L	L	L	L	L	L
9. Exposure measures.	L	L	L	L	L	L	L	L	L	L	L	L	L	L	L
10. Repeated exposure assessment.	L	L	L	L	L	L	L	L	L	L	L	L	L	L	L
11. Outcome measures.	L	L	L	L	L	L	L	L	L	L	L	L	L	L	L
12. Blinding of outcome assessors.	L	L	L	L	L	L	L	L	L	L	L	L	L	L	L
13. Follow-up rate.	L	L	L	L	L	L	L	L	L	L	L	L	L	L	L
14. Statistical analyses.	L	L	L	L	L	L	L	L	L	L	L	L	L	L	L

*L* low risk, *H* high risk, *NR* not reported

### Qualitative analysis (systematic review)

Table 2 summarizes the main features of the 15 included studies. The 15 studies were published between 2012 and 2020. All studies but one [29] were reported in the English language. Nine studies were performed by Asian/Eastern [17–20, 22–24, 27, 31] and six by Western [21, 25, 26, 28–30] authors. They were single-center or observational cohort studies. The total number of ^18^F-FDG PET/CT examinations was 232,568 identifying 4031 (1.7%) focal ^18^F-FDG TIs. The female-to-male ratio was approximately 3:1. Almost all patients were submitted for ^18^F-FDG PET/CT to stage non-thyroidal cancer. The FNA data according to BETHESDA was available in 2304 ^18^F-FDG TIs. The final histological diagnoses were reported in 41.3% of cases (618 cancers and 335 benign nodules).

**Table 2:** Main features of the 15 studies included in the present systematic review

First author [ref]	Year	Country	Total PET/CT performed	TIs	Age	F/M	SUVmax	Size (mm)	FNA classified according to Bethesda							Histology		
									Tot	I	II	III	IV	V	VI	Tot	Mal	Ben
Lee [17]	2012	Korea	327	17	56 ± 10	NA	4.3	NA	16	0	10	1	2	0	3	4	4	0
Kim [18]	2013	Korea	22,674	483	59 ± 11	NA	NA	NA	280	33	155	5	8	7	72	72	71	1
Choi [19]	2014	Korea	7914	171	56 ± 10	84/48	6.3	16	171	33	62	0	4	15	57	68	65	3
Lee [20]	2014	Korea	2368	64	58.8 ± 6.2	24/5	4.5	20	29	2	13	2	0	8	4	15	9	6
Jamsek [21]	2015	Slovenia	2840	148	61.2 ± 12.9	NA	4.2	NA	52	7	24	3	8	3	7	NA	NA	NA
Kim [22]	2015	Korea	18,172	374	58 ± 11	116/40	4	13	235	17	153	13	4	12	36	NA	NA	NA
Kim [23]	2015	Korea	23,462	493	50 ± 10	157/43	5.4	NA	200	14	116	12	6	13	39	72	49	23
Yoon [24]	2015	Korea	NA	87	59 ± 12	48/39	6.2	16.5	87	1	42	4	1	3	36	27	27	0
Hagenimana [25]	2017	Canada	40,914	304	NA	NA	NA	NA	159	36	63	14	23	15	8	46	25	21
Li [26]	2017	USA	NA	20	NA	12/8	NA	NA	20	0	6	2	8	2	2	11	9	2
Suh [27]	2017	Korea	96,942	1360	59 ± 11	1035/325	NA	NA	859	82	229	222	18	31	277	570	325	245
Thuillier [28]	2017	France	10,118	131	64.2 ± 11.6	55/45	NA	NA	60	16	24	5	12	1	2	19	10	9
De Guevara [29]	2020	Chile	5100	119	62	NA	NA	NA	58	1	20	4	4	8	21	NA	NA	NA
Kaliszewski [30]	2020	Poland	NA	56	56 ± 15	42/7	NA	NA	49	0	23	7	12	5	2	49	24	25
Kamakshi [31]	2020	India	1737	204	51	199/89	4.5	23	29	10	11	3	2	1	2	NA	NA	NA
*TOT*			232,568	4031		1772/649			2304	252	951	296	113	123	569	953	618	335

Age, SUVmax, and size findings were reported in the studies as mean or median

*TI* thyroid incidentaloma at PET/CT, *F* female, *M* male, *NA* not available

### Quantitative analysis (meta‑analysis)

The distribution of the 2304 ^18^F-FDG TIs according to BETHESDA was evaluated. Table 3 shows the pooled prevalence results. The most frequent cytologic category was benign (BETHESDA II) comprising 45% of ^18^F-FDG TIs. The malignant category (BETHESDA VI) was the second most common cytologic subcategory (19%). Inconsistency was found in all six categories. Publication bias was present in two categories.

**Table 3:** Pooled prevalence of the 2304 ^18^F-FDG TIs in the six Bethesda categories

TBS category	Prevalence (%), 95% CI	*I*^2^ (%)	Egger test (*p*)
I (ND)	10, 6–14	86.0	0.30
II (B)	45, 37–53	92.7	0.15
III (AUS/FLUS)	8, 3–13	94.6	0.11
IV (FN/SFN)	8, 5–12	88.2	0.001
V (SFM)	6, 4–9	69.8	0.03
VI (M)	19, 13–25	91.6	0.86

*TBS* the Bethesda system for reporting thyroid cytopathology, *ND* nondiagnostic, *B* benign, *AUS/FLUS* atypia of undetermined significance/follicular lesion of undetermined significance, *FN/SFN* follicular neoplasms/suspicious for follicular neoplasms, *M* malignant, *SFM* suspicious for malignancy, *CI* confidence interval, *I*^2^ inconsistency

In an attempt to explain the above heterogeneity, further analyses were performed. One sensitivity analysis included only those studies with more than 100 TIs [18, 19, 22, 23, 25, 27]. This analysis included 1904 ^18^F-FDG TIs. The prevalence in the BETHESDA subcategories was substantially unchanged (I 12%, II 47%, III 6%, IV 4%, V 6%, and VI 21%) and the inconsistency remained high (detailed data not shown). A second analysis was performed considering separately the nine studies from Asian/Eastern countries [17–20, 22–24, 27, 31] and the six studies from Western authors [21, 25, 26, 28–30] as shown in Table 4. There was a significantly different prevalence in the BETHESDA category IV between Asian/Eastern and Western studies. With this sub-analysis, no heterogeneity was identified in category IV of Asian/Eastern studies and in categories II, III, and V of the Western studies. Unfortunately, data on the frequency and presence or absence of HTL was not available to perform further specific analysis. Forest and funnel plots are included as supplemental material.

**Table 4:** Pooled prevalence of the ^18^F-FDG TIs in the six Bethesda categories in eastern and western studies compared with meta-analysis results for all thyroid nodules by Vuong et al. [13]

	Eastern studies			Western studies		
Studies, *n* (ref.)	9 [17–20, 22–24, 27, 31]		Vuong et al. [13]	6 [21, 25, 26, 28–30]		Vuong et al. [13]
Nodules, *n*	1906		69,907	398		75,159
TBS category	Prevalence %, 95% CI	*I*^*2*^	Prevalence %, 95% CI	Prevalence %, 95% CI	*I*^2^	Prevalence %, 95% CI
I (ND)	10, 6–14	82.2	12.6, 6.7–18.5	9, 2–20	90.4	11.9, 9.1–14.7
II (B)	48, 36–60	95.8	59.8, 51.6–67.9	40, 36–45	0	64.2, 60.0–68.4
III (AUS/FLUS)	7, 1–15	96.9	8.4, 5.5–11.4	9, 6–12	0	7.7, 5.1–10.2
IV (FN/SFN)^a^	2, 2–3	0.3	3.5, 1.9–5.1	19, 12–27	68.9	7.9, 5.7–10.1
V (SFM)	6, 4–8	70.4	4.3, 2.6–6.1	9, 5–13	37.1	3.3, 2.6–4.1
VI (M)	24, 18–31	86.4	10.9, 7.1–14.7	11, 4–21	86.2	4.9, 3.8–6.0

Here, the prevalence is pooled and differs from absolute prevalence since the figures also depend on the weight given to each study

*TBS* the Bethesda system for reporting thyroid cytopathology, *ND* nondiagnostic, *B* benign, *AUS/FLUS* atypia of undetermined significance/follicular lesion of undetermined significance, *FN/SFN* follicular neoplasms/suspicious for follicular neoplasms, *M* malignant, *SFM* suspicious for malignancy, *CI* confidence interval

^a^Indicates the categories in which there was a significantly different prevalence between Eastern and Western studies (i.e., the 95% CIs were not overlapping)

## Discussion

An evidence-based review detailing information on BETHESDA FNA subcategory outcomes in ^18^F-FDG focally avid nodules is not currently available in the literature. This systematic review with meta-analysis provides additional evidence-based data on the distribution of ^18^F-FDG TIs across the full range of BETHESDA cytologic subcategories, enabling more detailed consideration of clinical management in patients undergoing cytologic assessment for ^18^F-FDG TIs [8].

This study shows that FDG avid nodules show a relative excess of BETHESDA category IV and V FNA (see Table 4). Although the published studies included in this meta-analysis do not give specific information on the prevalence of Hurthle cell neoplasms, the finding of relatively higher frequencies of category IV and V FNA in thyroid TI implies that this is due to the underlying higher clinical ROM of PET avid thyroid nodules, in combination with a relative excess of HCN in nodules that are FDG avid which would typically fall in BETHESDA categories III and IV [14, 32–34].

Diagnostic assessment of ^18^F-FDG TIs represents a major challenge in clinical practice. The majority of focal ^18^F-FDG PET/CT avid thyroid nodules are benign. Unfortunately, we could not derive conclusions about potential relationships between SUVmax values and BETHESDA categories due to the paucity of data. Patients undergoing thyroid ^18^F-FDG PET/CT generally show more aggressive non-thyroidal comorbid tumors, hence investigation of a potential co-existent thyroid carcinoma may not be a therapeutic priority [10]. Although up to 35–40% of ^18^F-FDG TIs are malignant, this data is likely to be biased due to the inclusion of patients with non-thyroid tumors [10].^18^F-FDG-avid thyroid cancer is a malignancy said to be more aggressive than non^18^F-FDG-avid thyroid carcinoma [35].

This study does not address cancer prevalence among focal ^18^F-FDG TIs, rather it describes how a cytologic report can influence the clinical management of clinically suspected ^18^F-FDG TI patients as this information is important, especially for patients with more aggressive non-thyroid tumors. The results of this study show pooled rates for BETHESDA I non-diagnostic of 10%, BETHESDA II benign 45%, BETHESDA III AUS/FLUS 8%, BETHESDA IV FN/SFN 5%, BETHESDA V SFM 6%, and BETHESDA VI malignant of 19%. The frequency of BETHESDA V suspicious for malignancy and BETHESDA VI malignant FNA in ^18^F-FDG-avid thyroid nodules is much higher than the 7.4% published rate for Bethesda VI FNA and 3.7% for Bethesda V FNA shown in a recent meta-analysis of Western and Asian patients [13]. This study, therefore, indicates that ^18^F-FDG TIs have a higher ROM than that observed in the general population of thyroid nodules so requiring further investigation. A 45% of ^18^F-FDG TIs were cytologically assessed as benign, enabling the use of FNA as a rule-out test in these patients, most of whom have a more aggressive non-thyroidal cancer. Pooled prevalence data shows that overall, nearly two-thirds of ^18^F-FDG TIs have either a malignant or benign FNA report, which provides reassurance because the false positive and false negative rates of these categories are negligible (i.e., 2–3% and 0–3%, respectively) [12]. Overall, combining results from Western and Asian/Eastern studies, 13% of ^18^F-FDG TIs are indeterminate (8% BETHESDA III and 5% BETHESDA IV) although over one-quarter of the patients will have an inconclusive FNA based on Western studies whereas the figure for Asian/Eastern patients is much lower. Because the ROM for the indeterminate FNA categories approaches 30% [13], this represents a challenge for clinical practice. A relatively high number of BETHESDA IV FNA among patients with ^18^F-FDG TIs are oncocytic/Hürthle cell lesions [36, 37] although most studies do not separately record these lesions in published series.

Ultrasound assessment is known to be suboptimal for detection of follicular and oncocytic thyroid carcinomas as compared to papillary thyroid carcinoma; yet some thyroid cancers with poor prognosis, e.g., some follicular and oncocytic carcinomas, are typically classified as BETHESDA III or IV [38, 39]. Moreover, ^18^F-FDG avid thyroid cancer is a tumor that is generally more biologically aggressive [40–42]. The possibility of an indeterminate FNA result should be taken into account when requesting FNA in ^18^F-FDG TI. In addition, the presence of noninvasive follicular thyroid neoplasm with papillary-like nuclear features in Bethesda III and IV categories, as well as in the other categories, should also be considered [43, 44]. High heterogeneity was found in these findings. However, this heterogeneity can be explained according to the sub-analysis performed separating the studies published by Asian/Eastern and Western authors. As shown in Table 4, the heterogeneity was canceled in some cases, as there was a significant difference between Asian/Eastern and Western studies in BETHESDA category IV. The latter finding corroborates the results obtained by Vuong et al. [13]. However, in the Vuong et al. meta-analysis [13] the prevalence of category IV was 7.9% in Western and 3.5% in Asian/Eastern studies, while here we found 19% and 2%, respectively. The reasons for the published differences between Western and Asian/Eastern cytopathology practice are unclear. There may also be differences in nuclear thresholds for papillary thyroid carcinoma although data on this is lacking. Beyond any consideration of the ^18^F-FDG avid thyroid nodule risk (clinical information, biochemical tests, and US features), we should always keep in mind the context in which we are moving. Indeed, the extrathyroidal PET/CT findings should be carefully considered prior to the decision to undertake thyroid FNA. In this context, there are probably two optimal imaging scenarios to perform FNA biopsy: patients with complete remission of their non-thyroidal cancer and patients with ^18^F-FDG PET/CT findings suspected of a new diagnosis of metastatic thyroid cancer. In other terms, the higher the risk of finding thyroid cancer and the higher the likelihood of detection of a highly aggressive primary thyroid malignancy, the more appropriate the indication for FNA.

The pooled data of this meta-analysis could be affected by various biases which should be discussed. The studies included in this systematic review recorded 4031 focal ^18^F-FDG TIs while they reported the results of 2304 (57.1%) FNAs. It is unclear whether patients were managed according to specific clinical features, US-related risk, SUVmax value, or other characteristics associated with the cancers which indicated PET/CT (i.e., selection bias). Whether the cytopathologists were influenced by the FNA indication (i.e., ^18^F-FDG TI in an oncological patient) is also not reported. The studies report the results of a retrospective review of single-center series of patients. The PET/CT systems utilized were different with different sensitivity and resolutions. Finally, a high statistical heterogeneity among the included studies was found although this heterogeneity can be partially explained after a sub-analysis of the two groups of Asian/Eastern and Western studies.

## Conclusions

This meta-analysis shows for the first time that two in three of ^18^F-FDG TIs undergoing FNA have a malignant (BETHESDA VI) or benign (BETHESDA II) cytology. The remaining approximately one-quarter of cases have an indeterminate FNA and the remainder are non-diagnostic FNA. A significant difference between Asian/Eastern and Western studies is present in the prevalence of the indeterminate category IV. Thyroidologists should be aware of this data to enable better management of patients, especially when an aggressive non-thyroid cancer is present. This evidence-based data suggests guiding clinical decision-making according to the patient’s clinical context, including the indication for FNA and extra-thyroidal findings of ^18^F-FDG PET/CT.

## Supplementary information

Supplementary information
