# Supplementary material for: The dilemma of 18F-FDG PET/CT thyroid incidentaloma: what we should expect from FNA. A systematic review and meta-analysis
Source: Endocrine. 2021 Mar 24;73(3):540–9. doi: 10.1007/s12020-021-02683-4 (PMC8325664; doi:10.1007/s12020-021-02683-4)

BETHESDA I

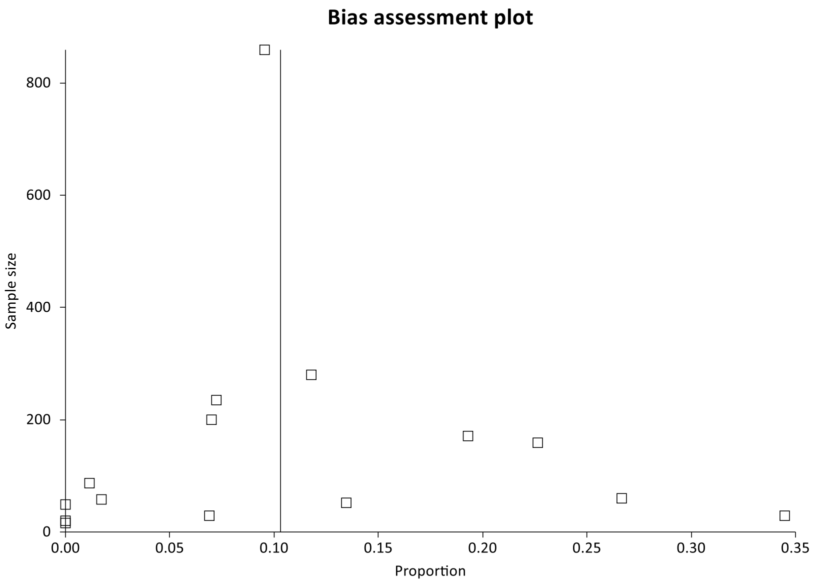

Bias assessment plot
Sample size
800
600
400
200
0
0.00
0.05
0.10
0.15
0.20
0.25
0.30
0.35
Proportion

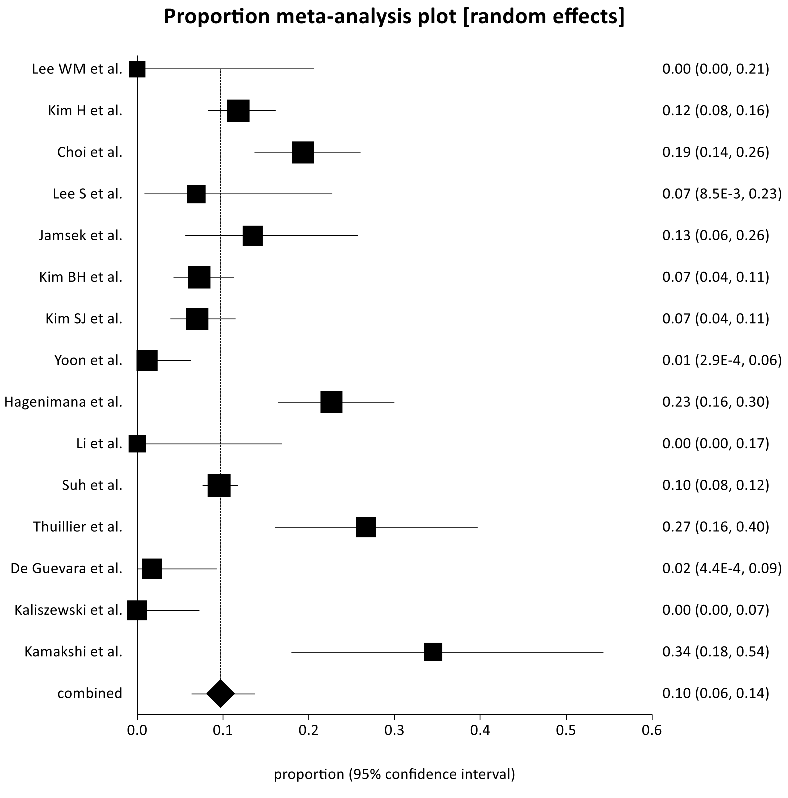

Proportion meta-analysis plot [random effects]
Lee WM et al. 0.00 (0.00, 0.21)
Kim H et al. 0.12 (0.08, 0.16)
Choi et al. 0.19 (0.14, 0.26)
Lee S et al. 0.07 (8.5E-3, 0.23)
Jamsek et al. 0.13 (0.06, 0.26)
Kim BH et al. 0.07 (0.04, 0.11)
Kim SJ et al. 0.07 (0.04, 0.11)
Yoon et al. 0.01 (2.9E-4, 0.06)
Hagenimana et al. 0.23 (0.16, 0.30)
Li et al. 0.00 (0.00, 0.17)
Suh et al. 0.10 (0.08, 0.12)
Thuillier et al. 0.27 (0.16, 0.40)
De Guevara et al. 0.02 (4.4E-4, 0.09)
Kaliszewski et al. 0.00 (0.00, 0.07)
Kamakshi et al. 0.34 (0.18, 0.54)
combined 0.10 (0.06, 0.14)
0.0
0.1
0.2
0.3
0.4
0.5
0.6
proportion (95% confidence interval)

BETHESDA II

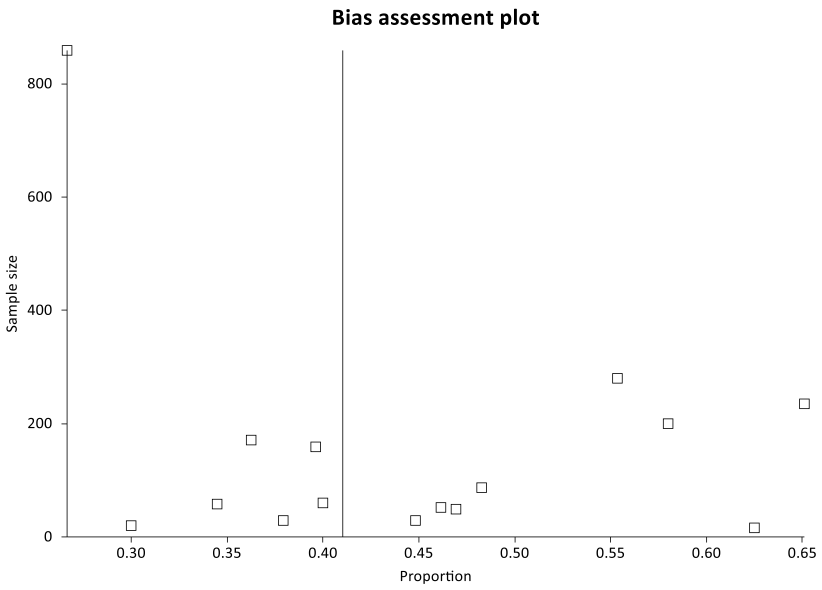
Bias assessment plot
Sample size
800
600
400
200
0
0.30
0.35
0.40
0.45
0.50
0.55
0.60
0.65
Proportion

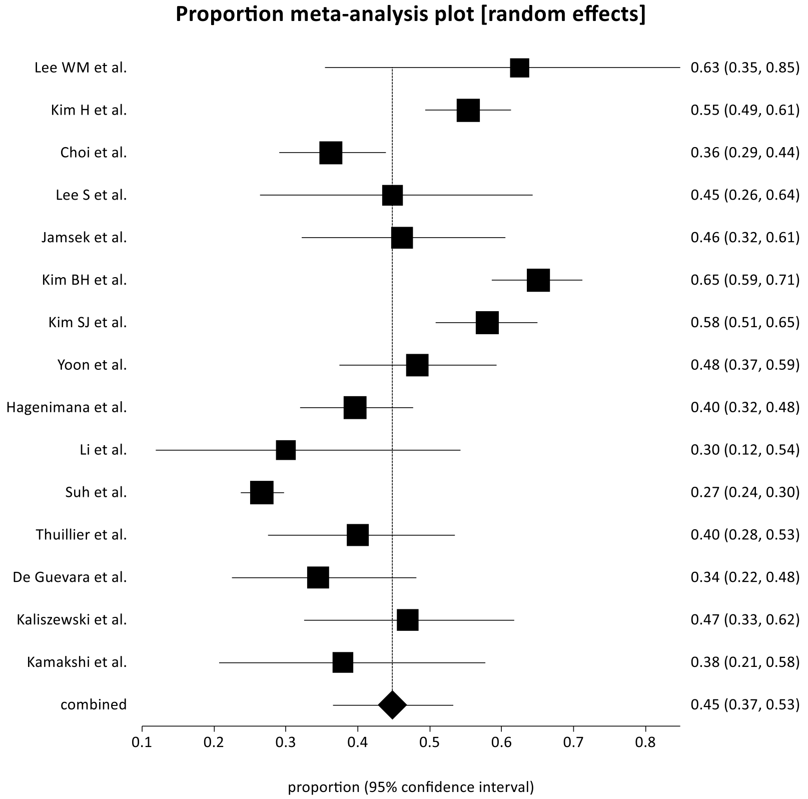
Proportion meta-analysis plot [random effects]
Lee WM et al.
0.63 (0.35, 0.85)
Kim H et al.
0.55 (0.49, 0.61)
Choi et al.
0.36 (0.29, 0.44)
Lee S et al.
0.45 (0.26, 0.64)
Jamsek et al.
0.46 (0.32, 0.61)
Kim BH et al.
0.65 (0.59, 0.71)
Kim SJ et al.
0.58 (0.51, 0.65)
Yoon et al.
0.48 (0.37, 0.59)
Hagenimana et al.
0.40 (0.32, 0.48)
Li et al.
0.30 (0.12, 0.54)
Suh et al.
0.27 (0.24, 0.30)
Thuillier et al.
0.40 (0.28, 0.53)
De Guevara et al.
0.34 (0.22, 0.48)
Kaliszewski et al.
0.47 (0.33, 0.62)
Kamakshi et al.
0.38 (0.21, 0.58)
combined
0.45 (0.37, 0.53)
0.1
0.2
0.3
0.4
0.5
0.6
0.7
0.8
proportion (95% confidence interval)

BETHESDA III

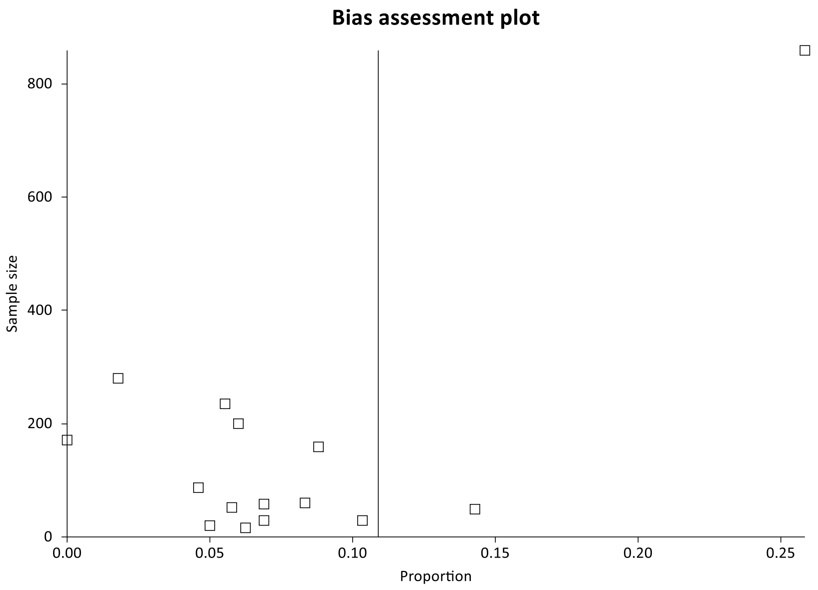

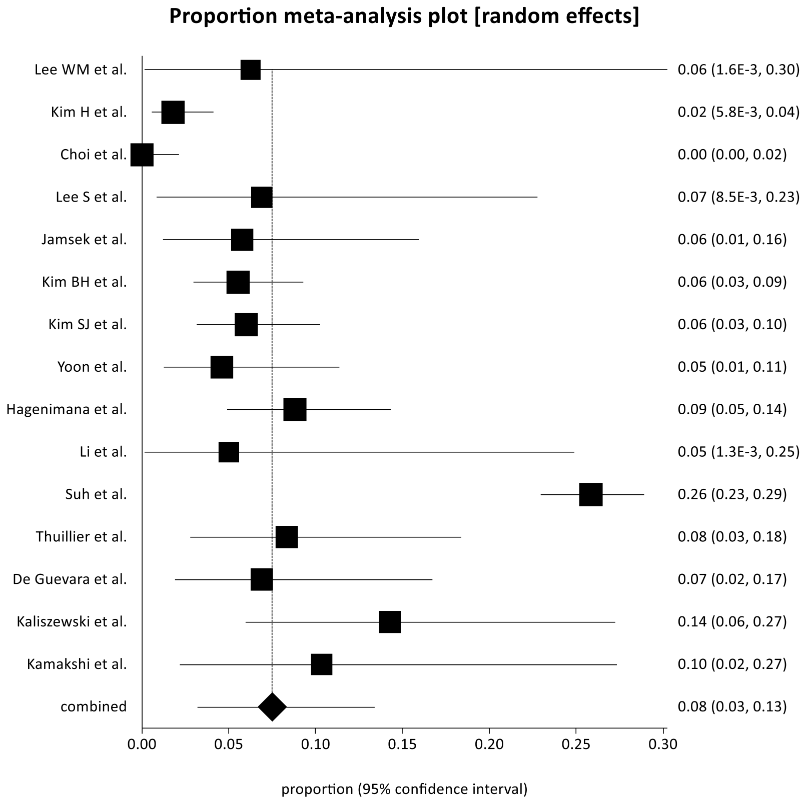

BETHESDA IV

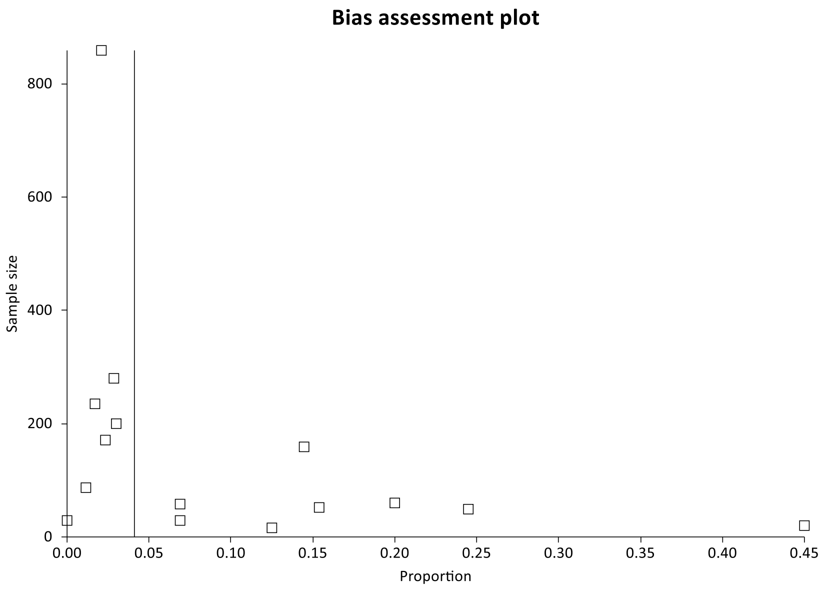
Bias assessment plot
Sample size
800
600
400
200
0
0.00
0.05
0.10
0.15
0.20
0.25
0.30
0.35
0.40
0.45
Proportion

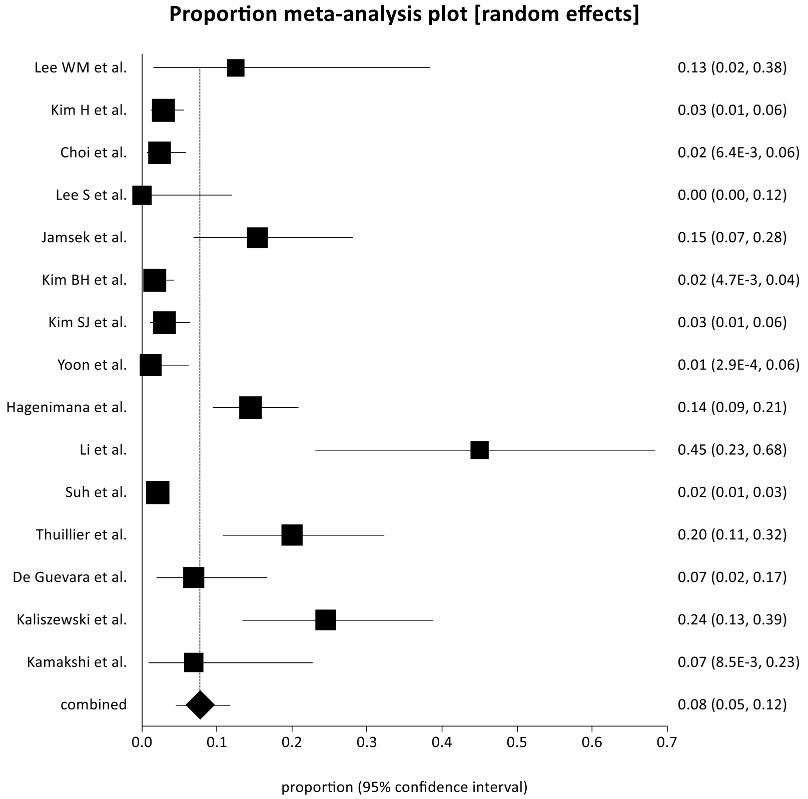
Proportion meta-analysis plot [random effects]
Lee WM et al.
0.13 (0.02, 0.38)
Kim H et al.
0.03 (0.01, 0.06)
Choi et al.
0.02 (6.4E-3, 0.06)
Lee S et al.
0.00 (0.00, 0.12)
Jamsek et al.
0.15 (0.07, 0.28)
Kim BH et al.
0.02 (4.7E-3, 0.04)
Kim SJ et al.
0.03 (0.01, 0.06)
Yoon et al.
0.01 (2.9E-4, 0.06)
Hagenimana et al.
0.14 (0.09, 0.21)
Li et al.
0.45 (0.23, 0.68)
Suh et al.
0.02 (0.01, 0.03)
Thuillier et al.
0.20 (0.11, 0.32)
De Guevara et al.
0.07 (0.02, 0.17)
Kaliszewski et al.
0.24 (0.13, 0.39)
Kamakshi et al.
0.07 (8.5E-3, 0.23)
combined
0.08 (0.05, 0.12)
0.0
0.1
0.2
0.3
0.4
0.5
0.6
0.7
proportion (95% confidence interval)

BETHESDA V

Bias assessment plot

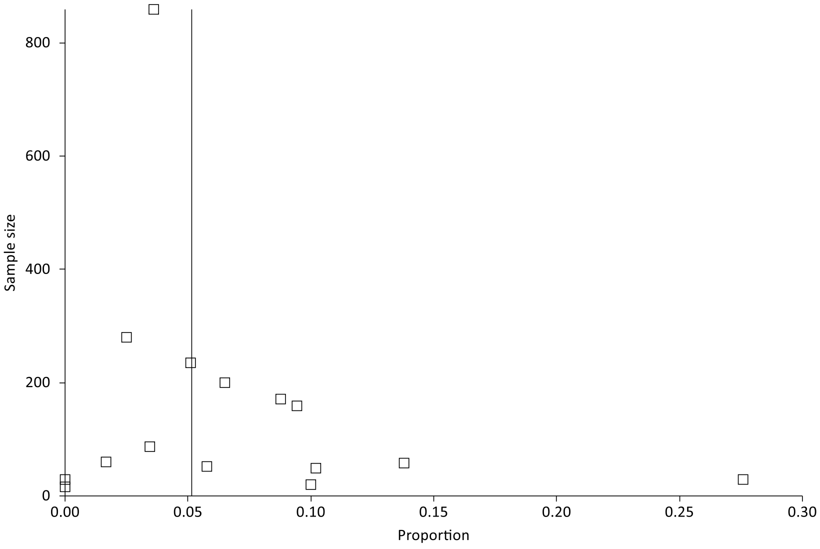

Proportion meta-analysis plot [random effects]

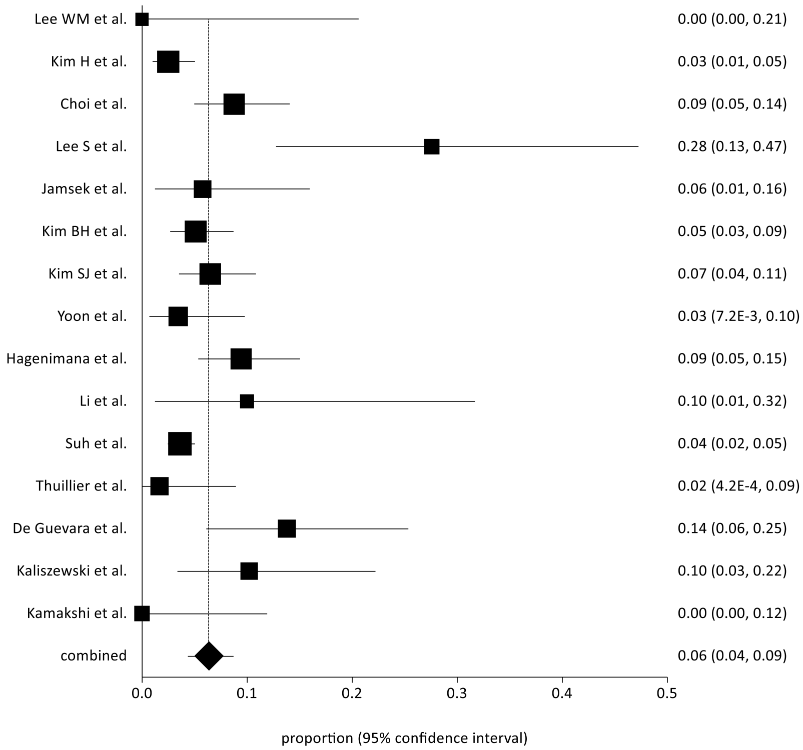

BETHESDA VI

Bias assessment plot

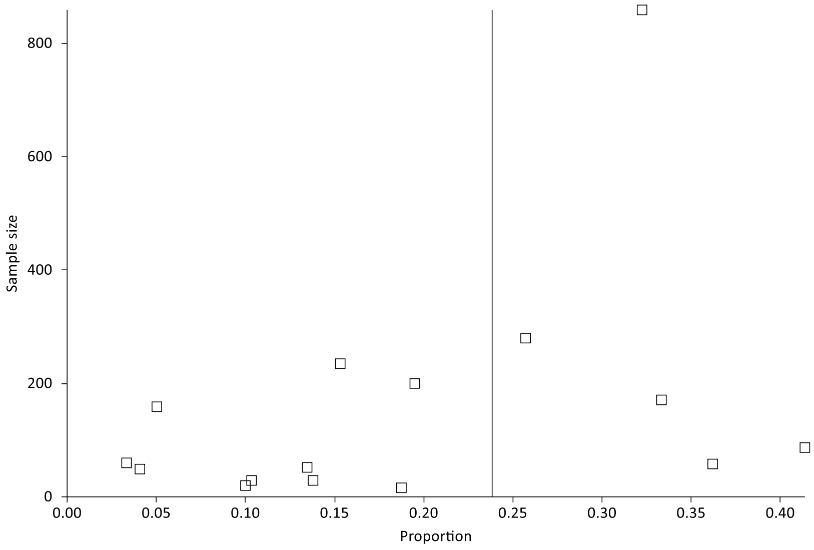

Proportion meta-analysis plot [random effects]

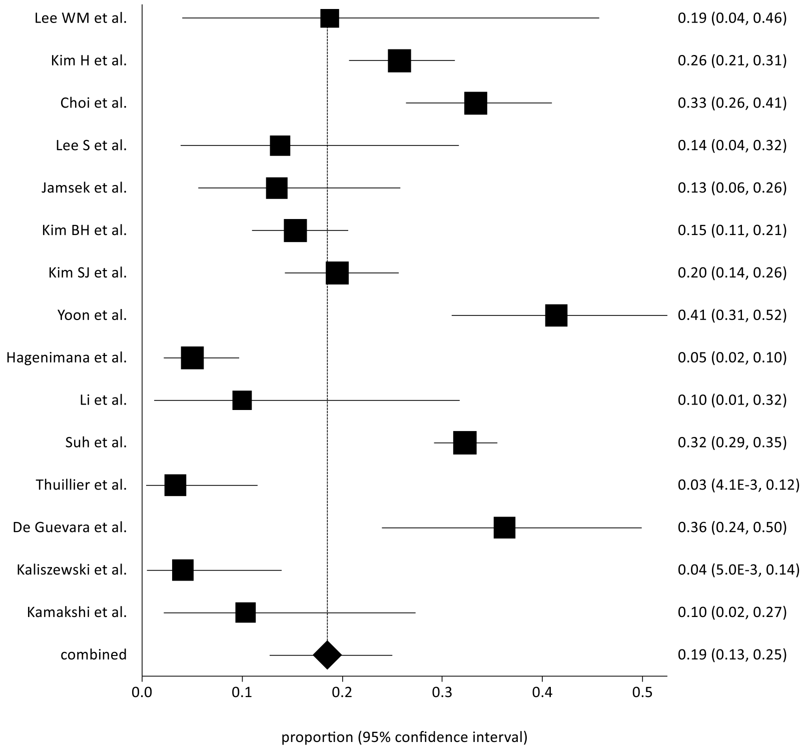

Supplement: Supplementary file 1 — Supplementary information [file 12020_2021_2683_MOESM1_ESM.pdf]
